# Sex-Specific Human Milk Composition: The Role of Infant Sex in Determining Early Life Nutrition

**DOI:** 10.3390/nu10091194

**Published:** 2018-09-01

**Authors:** Laura Galante, Amber M. Milan, Clare M. Reynolds, David Cameron-Smith, Mark H. Vickers, Shikha Pundir

**Affiliations:** 1 Liggins Institute, the University of Auckland, Auckland 1142, New Zealand; l.galante@auckland.ac.nz (L.G.); a.milan@auckland.ac.nz (A.M.M.); c.reynolds@auckland.ac.nz (C.M.R.); d.cameron-smith@auckland.ac.nz (D.C.-S.); m.vickers@auckland.ac.nz (M.H.V.); 2 Riddet Institute, Massey University, Palmerston North 4442, New Zealand; 3 Food & Bio-Based Products Group, AgResearch, Palmerston North 4442, New Zealand

**Keywords:** human milk, sex-specificity, infant growth, early life nutrition, postnatal outcomes, breastfeeding

## Abstract

Male and female infants respond differentially to environmental stimuli, with different growth and neurodevelopmental trajectories. Male infants are more likely to be disadvantaged when subjected to adversity and show a higher risk of perinatal complications. However, the underlying causes of this sex-bias are not well defined and optimising the early life nutritional care may be necessary to minimise the “male disadvantage” that may be experienced early in life. Experimental models have demonstrated that animal milk composition differs according to offspring sex, suggesting that the tailoring of early life nutrition may be one mechanism to maximise health protection and development to infants of both sexes. However, evidence for a sex-specificity in human milk composition is limited and conflicting, with studies documenting higher milk energy content for either male or female infants. These data show sex differences, however, there has been limited compositional analysis of the current data nor strategies proposed for how sex-specific compositional differences in early life nutrition may be used to improve infant health. The present narrative review highlights that an improved understanding of sex-specific human milk composition is essential for promoting optimal infant growth and development.

## 1. Introduction

Human milk (HM) is the gold standard for infant nutrition, providing the necessary building blocks required for postnatal growth and development [1,2]. The biochemical composition of HM is highly dynamic and varies between mothers and within the same mother throughout the different stages of lactation [3,4,5,6] and within a feed, such that foremilk and hindmilk have differing biochemical compositions [7]. HM composition changes most notably during the transition from colostrum to mature milk, with the intermediate of transitional milk, as lactation proceeds [8]. However, the dynamic regulation of HM composition is also impacted by maternal, environmental, and infant factors. Changes in maternal diet significantly influence HM macronutrient composition, particularly within the lipid fraction. This is evident in mothers consuming a western-style diet, where high dietary consumption of omega-6 is directly reflected in the HM lipid profile [9]. Further, maternal BMI has also documented impacts on HM lipid profile with different fatty acid profile between obese and lean mothers [10,11]. Along with macronutrient composition, non-nutritive milk bioactives are also impacted by maternal factors such as stress [12] and BMI [13]. HM bioactives, such as growth [14] and satiety factors [15,16], and different classes of hormones, such as steroids [17], play important roles in human physiology, including the regulation of energy intake [18,19] and somatic growth [20,21]. Interestingly, most of these bioactive compounds have been reported to follow sex-specific secretion pathways in adults, children [22,23], and in cord blood [24,25], yet, little is known about their sex-specific distribution in HM.

Despite international recognition of the importance of breastfeeding, not all infants can be exclusively breastfed. Some mothers indeed encounter challenges in establishing and/or maintaining lactation over the first six months of life [26,27,28,29], which results in early weaning or formula-feeding for their infants. Nutritional and hormonal exposures during the first 1000 days of life are known to be important for both short- and long-term health outcomes [30]. In this context, the observation that male and female infants have a sexually-dimorphic response to their early nutritional environment [31] suggests that there are sex-specific requirements during this early phase of life for optimal growth and development. Yet, there are currently no practice guidelines establishing different nutritional strategies or requirements for male and female infants [32]. For this reason, a better understanding of whether male and female infants receive different nutritional and hormonal intakes through maternal HM would provide important information around the potential need to tailor nutritional strategies for clinical and community settings in a sex-specific manner. 

The present work constitutes a narrative review of the existing evidence regarding sex-specificity in HM composition in relation to sex-specific health outcomes such as the higher perinatal risk of mortality and morbidity observed in male neonates.

## 2. The “Male Disadvantage”: A Consequence of Sex-Specific Requirements?

Differences in perinatal outcomes between males and females have been recognised since the 1970’s as the “male disadvantage” [33]. The concept arose from the observation that male infants in the United States had a higher risk of neonatal mortality compared to females, and that this was not related to specific disease processes. Newborn males are known to be more vulnerable than females to postnatal complications including respiratory distress syndrome [34], neonatal anaemia and mineral deficiencies, particularly in high-risk populations such as low birthweight (LBW) infants [35]. Stevenson et al., later examined the “male disadvantage” to understand whether technological advances in neonatal care, such as improved ventilation, enhanced surfactant therapies and administration of antenatal steroids, would improve outcomes in male infants. However, despite an overall decrease in mortality following the implementation of these strategies, a sex-bias was still observed in male infants [36]. 

At present, sex-specific differences in perinatal health outcomes, including neurological, metabolic and respiratory complications, with males having higher risk for poorer health [37,38], remain of concern as a cause of higher mortality and morbidity in male newborns. While the main cause for this bias is unknown, the observation that female and male offspring react differently to early life nutritional stimuli [39,40] may be a crucial factor in our understanding of the mechanisms underlying these differences in postnatal outcomes. Research carried out on ovine model has shown that offspring of different sexes respond differently to a standard supplemental nutrition [41]. When newborn lambs were randomised to receive maternal milk and either milk fortifiers or water for two weeks they showed differential outcomes compared to same-sex controls, with supplemented males displaying increased insulin in response to the intravenous glucose tolerance test (IVGTT) received at four months. Conversely, insulin was lower after the IVGTT in supplemented females compared to female controls. Divergent responses to infant feeding have also been documented in humans. Lucas et al., demonstrated that in humans, male preterm neonates were more responsive than females to preterm formula with higher protein, energy content and micronutrients to meet higher preterm requirements. These infants displayed higher Bayley’s test scores, indicative of improved neurodevelopment, at 18 months of age, in comparison to male infants receiving a standard term formula [31]. Given the evidence of sex-specific infant outcomes based on differences in early life nutritional support, an improved understanding of the innate compositional differences in HM produced for these infants is essential to establishing requirements and guidelines for supplemental support.

## 3. Sex-Specific Composition of Maternal Milk

Evidence from animal models (Table 1) suggests that infant sex is a predictive determinant of maternal milk composition. Primate [41,42,43] and bovine [44] models have demonstrated that mothers produce different milk for male and female singleton offspring. For example, macaque (*Macaca mulatta*) mothers of male offspring produced a lower volume of milk but with a higher energy content, whereas mothers of female offspring had greater volume of milk which was less energy-dense, resulting in similar total energy content of the milk produced for both sexes [42]. Yet, milk produced for female macaque offspring had higher calcium content [41]. In a bovine model, mothers have been shown to produce considerably more milk and of higher energy content for their female offspring [44]. Evidence of sex-specific milk production has also been reported for other ruminants, as well as marsupials. Wild eastern kangaroos (*Macropus giganteus*) [45] and Tammar wallabies (*Macropus eugenii*) [46] were found to produce milk with higher protein for male offspring but same energy content and volume for offspring of different sex. Similarly, Iberian red deer (*Cervus elaphus hispanicus*) mothers were observed to produce greater yields of milk with higher energy content for male calves, reflected by higher protein, fat and lactose content [47]. While these studies provide evidence that the sex of offspring has an impact on the nutritional composition of maternal milk across very different *taxa*, the range of milk-borne compounds analysed in each study is limited and no information is available around hormonal concentration in milk produced for male and female offspring. Furthermore, the mechanisms driving sex-specific milk synthesis are currently unclear and need to be investigated in order to enable a better understanding of the phenomenon.

Despite data derived from experimental models, there remains a paucity of data around the potential for sex-specificity in HM composition in regards to nutrients and bioactive compounds. The potential for a sex-specificity has however been suggested by clinical research on twins [48]. In this setting, opposite-sex twins, that receive HM which cannot simultaneously be tailored for both sexes, have been hypothesised to have different outcomes in comparison with same-sex twins in regards to postnatal growth [48]. Kanazawa and Segal observed indeed that breastfed opposite-sex twins were on average 1 inch shorter and 12 pounds lighter than same-sex twins during adolescence and early adulthood. This may suggest that the sex-specificity of HM might have a role in the early growth of the infant and exerts effects that persist throughout the life course of the individual.

Nonetheless to date only six human studies have reported on the relationship between HM composition and infant sex [49,50,51,52,53,54]. These studies primarily focused on macronutrient profile and energy content of HM and reported conflicting relationships in regard to infant sex. In particular, one study on Filipino mothers found no association between HM composition and infant sex [52]. Another study reported that American mothers of male infants produce HM with a greater energy content [49]; whereas, other research groups found that HM was higher in energy for female infants in Korean and Kenyan mothers [50,51]. Interestingly, the study on Kenyan mothers reported a conditional association between female sex and higher fat content in HM, that was displayed only by mothers with low socioeconomic status [50], supporting the Trivers-Willard hypothesis of unequal parental investment between female and male offspring depending on maternal condition [55]. Mirroring experimental models, HM for female infants in Iraqi mothers was found to have a higher calcium content; yet phosphorus content and total volume were reduced relative to the HM produced for male infants [53]. However, these studies reported several limitations and were conducted in different contexts and with very different sampling and analytical methods, as summarized in Table 2, which likely explain the disparity in findings. Nevertheless, despite being limited by collection methods, Fujita’s study [50] highlights the fact that HM composition is affected not only by multiple factors but also by the interactions between factors. A similar conditional association of the infant sex with HM composition has been reported by Fields et al., while looking at the influence of maternal BMI on HM composition [54]. Here, similarly to what was observed in Kenyan mothers, where fat content in HM was conditionally altered for female infants by the socioeconomic status of the family, Fields et al., found that insulin and leptin concentrations in maternal HM were the highest for mothers of females only when maternal BMI was very high [54].

Importantly, there is emerging research demonstrating a sex-specific differential effect of animal milk bioactives on offspring outcomes. As an example, elevated cortisol in the milk of lactating macaques has been found to correlate with a more nervousness and less confident temperament, impacting male offspring to a greater extent than female [56]. However, existing evidence supporting the idea of a sex-specific response to the nutrition received during early life (Table 3) is limited. The available findings often relate to subjects at risk, such as those born preterm or small for gestational age [31,57] and only assess the effect of the nutrition received immediately after birth [40]. Furthermore, in most of these studies the compositional analysis of maternal milk is not included, preventing the possibility of any association between specific milk-borne compounds and sex-specific response. This suggests the need for further research to fully understand the impact of nutritional and hormonal intakes during this sensitive period on sex-specific infant growth and development.

.

## 4. Conclusions

Evidence to date suggests that infants of different sex may have different nutritional and hormonal requirements. This underpins a need for sex-specific nutritional strategies for male and female infants, in order to optimise their growth and development. Experimental and limited clinical observations that maternal HM composition changes dependent on male or female sex, support this hypothesis. However, these reported sex-specific differences in HM composition require validation and clarification in additional human cohorts. These studies should aim to describe sex-specific differences in HM composition across the course of lactation and correlate differences with sex-specificity of infant outcomes, including sex-specific growth trajectories and sex-specific morbidity risk.

## Figures and Tables

**Table 1:** Overview of animal studies on sex-specificity in maternal milk.

Species		Sample Size	Offspring Age at Collection	Sex-Specific Difference	Study
**Primates**	Rhesus Macaque (*Macaca mulatta*)	106	3–4 months	↑ energy and ↑ fat for males	Hinde, 2007 [43]
		114 (62 F, 52 M)		↓ volume ↑ energy density for males	Hinde, 2009 [42]
		104 (61 F, 43 M)		↑ calcium for females	Hinde et al., 2013 [41]
**Ruminants**	Holstein breed of cow (*Bos Taurus*)	113,750 (data from lactation records)	not reported	↑ volume for females	Hinde et al., 2014 [44]
	Red deer (*Cervus elaphus hispanicus*)	91 (44 M, 47 F)	2, 6, 10 and 14 weeks	↑ volume, ↑ protein, ↑ fat and ↑ lactose for males	Landete-Catillejos et al., 2005 [47]
**Marsupials**	Kangaroo (*Macropus giganteus*)	91	6–10 months	↑ protein for males	Quesnel et al., 2017 [45]
	Wallaby (*Macropus eugenii*)	2 milking sessions: 15 in July (6 M, 9 F), 11 in October (4 M, 7 F)	4–8 months	↑ protein for males	Robert and Braun, 2012 [46]

F, female offspring; M, male offspring; ↑, higher compared to the opposite sex; ↓, lower compared to the opposite sex.

**Table 2:** Summary of human research on sex-specific human milk (HM) composition.

Study	Country	Sample Size	Collection Methods	Infant Age at Collection	Findings	Limitations
Yahya et al., 2009 [53]	Iraq	109 (52 M, 57 F)	Foremilk collected	Not specified	↑ calcium for females, ↑ volume and phosphorus for males	Composition not representative of hindmilk
Powe et al., 2010 [49]	United States of America	25	Breast was emptied by pump expression (mother’s pump or study pump).	2–5 months	↑ energy content (derived by carbohydrates, protein and lipid content) for males	Small sample size, inconsistent sampling time and use of instruments for milk collection, inconsistency of stage of lactation at sampling
Hahn et al., 2016 [51]	South Korea	478 (244 M, 234 F)	Sample collected during day time	0–3 months	↑ carbohydrate and energy content for females	Inconsistent sampling time, Absence of information on maternal diet and anthropometry, inconsistency of stage of lactation at sampling
Fujita et al., 2012 [50]	Kenya	83 (47 M, 36 F)	Foremilk collected in the morning by manual expression	Not specified	↑ fat for females only in mothers with a low socioeconomic status	Composition not representative of hindmilk
Quinn, 2013 [52]	Philippines	103 (52 M, 51 F)	Sample collected in the morning by manual expression after mother nursed the infants for approximately 3 minutes	0–18 months	No significant differences were found between male and female infants in HM composition	Composition not representative of foremilk, inconsistency in stage of lactation at sampling
Fields et al., 2017 [54]	Australia	37(16 M, 21 F)	Breast emptied by pump expression	1 and 6 months	↑ insulin and leptin for females born to obese mothers	Small sample size

F, female infant; M, male infant; ↑, higher compared to the opposite sex.

**Table 3:** Overview of animal and human studies on sex-specific response to early life nutrition.

		Sample Size	Nutrition	Sex-Specific Response	Limitations	Study
Animal	Sheep (*Ovis aries*)	42	Ewe’s milk + milk fortifiers vs. Ewe’s milk + water	↑ Insulin response in supplemented males, ↓ insulin response in supplemented females	Absence of direct estimates of milk composition and feeding behaviours	Jaquiery et al., 2016 [40]
	Rhesus Macaque (*Macaca mulatta*)	108	Maternal milk with ↑ cortisol	Males were more nervous and less confident	Absence of other hormonal measurements and lack of sensitive methodology to measure cortisol in milk	Hinde et al., 2015 [56]
Human		752	HM vs. FM	Breastfed same-sex twins were on average 1 inch taller and 12 pounds heavier than opposite-sex twins	Absence of HM compositional analyses and subjects’ body composition	Kanazawa and Segal, 2017 [48]
		424	Preterm FM vs. term FM	Preterm males fed preterm formula had ↑ Bayley’s test scores at 18 months	Absence of full term control group	Lucas et al., 1990 [31]
		76	Standard vs. high-nutrient diet	Male adolescents fed with high-nutrient diet after preterm birth had larger caudate nucleus volume and verbal IQ	Small sample size, absence of a full term control group	Isaacs et al., 2008 [57]

↑, higher compared to control and opposite sex; ↓, lower compared to control and opposite sex; IQ, intelligence quotient; FM, formula milk.
